# Bevacizumab for the treatment of non-small cell lung cancer patients with synchronous brain metastases

**DOI:** 10.1038/s41598-019-54513-3

**Published:** 2019-11-28

**Authors:** Mustafa S. Ascha, Jacqueline Fang Wang, Priya Kumthekar, Andrew E. Sloan, Carol Kruchko, Jill S. Barnholtz-Sloan

**Affiliations:** 1 Center for Clinical Investigation, Department of Population and Quantitative Health Sciences, Case Western Reserve University School of Medicine, Cleveland, Ohio USA; 2 Case Western Reserve University School of Medicine, Cleveland, Ohio USA; 3 Northwestern University Feinberg School of Medicine, Department of Neurology, Evanston, Illinois USA; 4 Department of Neurosurgery, University Hospitals Cleveland Medical Center, Seidman Cancer Center, and the Case Comprehensive Cancer Center, Cleveland, Ohio USA; 5 Central Brain Tumor Registry of the United States, Hinsdale, Illinois USA; 6 Department of Population and Quantitative Health Sciences, Case Western Reserve University School of Medicine, Cleveland, Ohio USA

**Keywords:** Targeted therapies, Metastasis, Molecularly targeted therapy

## Abstract

Bevacizumab is FDA-approved in the treatment of primary brain tumors, but its efficacy in patients with brain metastases could be better-studied. This study examines a population of non-small cell lung cancer (NSCLC) patients with synchronous brain metastases to identify predictors of the decision to use bevacizumab and survival following bevacizumab treatment. Primary cancer registry data were used to determine which NSCLC patients diagnosed in the years 2010 through 2012 had synchronous brain metastases at the time of diagnosis, and Medicare claims used to identify a population of patients treated with bevacizumab. Record of bevacizumab treatment was found for 81 and 666 patients with and without brain metastases, respectively. After adjusting for clinical and demographic characteristics, bevacizumab was associated with 0.88 times the hazard of mortality in the elderly NSCLC population (95% CI: 0.81–0.96, p: 0.003) and a corresponding hazard ratio of 0.75 in the population of elderly NSCLC patients with synchronous brain metastases (95% CI: 0.59–0.96, p: 0.020). Bevacizumab may benefit NSCLC patients with synchronous brain metastases more than it does patients without intracranial disease, possibly as a result of its multiple potential mechanisms of action simultaneously inhibiting angiogenesis and minimizing vasogenic edema.

## Introduction

Bevacizumab, a monoclonal antibody that inhibits angiogenesis by neutralizing Vascular Endothelial Growth Factor A (VEGF-A), improves PFS for GBM and is FDA approved for this common primary brain tumor^1,2^. Whereas VEGF-A expression normally directs angiogenesis in response to trauma, exercise, or for vasculogenesis in embryonic development, increased expression in tumors is pathological and simply increases the nutrient supply to tissue that will use it to grow exponentially. VEGF-A is the primary VEGF in its sub-family of platelet-derived growth factors, therefore this therapy directly impedes tumor growth by blocking a crucial oncogenic signal.

Bevacizumab reduces the increase in vascular permeability that is associated with VEGF expression and consequently helps relieve patients of the potentially serious morbidity and symptoms that accompany peritumoral edema^3^. This is particularly important in the CNS as edema can lead to increased intracranial pressure in the fixed volume of the cranial vault, which has potentially fatal consequences. Intracranial edema is frequently managed using corticosteroids, but bevacizumab both targets tumor growth mechanisms and simultaneously relieves symptoms, potentially sparing a patient of the effects of corticosteroids while also addressing the underlying disease^4–7^.

Though several studies have addressed the *safety* of bevacizumab treatment for brain metastases (BM), its efficacy for this purpose is less well-explored: one meta-analysis reports that, of 57 anti-VEGF treatment studies, 76% explicitly stated the presence of central nervous system metastases was among exclusion criteria, and only four studies reported on its use treating patients with BM^8–12^. As a result, researchers of BM in NSCLC suggest caution when considering bevacizumab for patients with active BM^13^ until ongoing clinical trials of this subject yield more conclusive evidence^14^.

For research that relies on analyses of healthcare claims, the dearth of studies regarding bevacizumab for BM can be explained by the limited accuracy of secondary cancer diagnosis codes. In 2016, however, the Surveillance, Epidemiology, and End-Results (SEER) program released its own data regarding diagnosis of BM during primary cancer staging workup; these high-fidelity cancer registry data may then be related to healthcare claims, further opening the door to large-scale analysis of BM treatment and outcomes.

This study identifies NSCLC patients with and without SBM treated with bevacizumab using Medicare claims data and evaluates the survival benefit of treatment with respect to primary cancer characteristics available from SEER, while further adjusting for treatment with several commonly-used chemotherapeutic agents. The resulting analysis offers insight into the treatment patterns and efficacy of bevacizumab among Medicare patients with NSCLC SBM.

## Materials and Methods

This study was approved as exempt of review by the University Hospitals Cleveland Medical Center Institutional Review Board under study number “EM-17–05.”, reviewed and approved by the SEER-Medicare committee as sharing no identifying information and preserving both privacy and confidentiality, and performed and reported in accordance with STROBE guidelines.

### Dataset

The SEER program of the National Cancer Institute collects cancer data from 18 sites throughout the United States, representing about 27% of the population. SEER data may be linked to Medicare claims for further investigation, thus enabling us to identify the use of monoclonal antibodies in subjects aged 65 years or older. SEER data include an element reflecting BM diagnoses made at the same time as primary cancer diagnosis (“synchronous”, or SBM), and are directly abstracted by cancer registrars from medical records.

Five types of claim files offered as part of SEER-Medicare were used for this project: Part A inpatient claims (MEDPAR), carrier claims (NCH), outpatient (OUTSAF), durable medical equipment (DME), and Part D drug prescription files. Each record in these files contains a date of service, International Classification of Diseases, Ninth revision, Clinical Modification (ICD-9-CM) diagnosis codes, Current Procedural Terminology (CPT), and Healthcare Common Procedure Coding System (HCPCS) procedure codes that were used to identify treatment and BM diagnoses.

Age at diagnosis was reported as age groups 65–70, 71 to 75, 76 to 80, and over 80 years. Race was examined in terms of three categories: White Non-Hispanic, Black, and Other. The histology of lung cancer was categorized into non-adenocarcinoma and adenocarcinoma histologies. Derived American Joint Committee on Cancer staging data were used to characterize diagnoses at stages I through IV, with A and B subcategories for stages I through III.

### Data-derived definitions

Healthcare Common Procedural Coding System (HCPCS) codes indicating bevacizumab use in non-small-cell lung cancer patients were identified in Medicare claims spanning 2007 through 2014 for patients whose primary cancer was diagnosed in the years 2010 through 2012, and included codes S0116, J9035, C9257, C9214, and Q2024^15^; this range of years of claims was selected to account for potential errors or delays in processing claims. Because bevacizumab has a half-life ranging on the order of weeks, record of only one infusion was sufficient for a patient to have been considered as treated with bevacizumab. Medications listed in the NCI’s chemotherapy lookup tables are included as adjustors, where potential medications must have been used in at least 11 patients diagnosed with brain metastases.

### Population

Each case was included only if it was the first cancer diagnosis for that patient, and lung cancers were selected on the basis of WHO site recoding (code: 22030^16^). Because bevacizumab is used for non-small-cell lung cancers, patients with small-cell or neuroendocrine lung cancer according to ICD-O-3 histology codes were excluded from this analysis (exclusion codes: 8002, 8041–8045)^17^. Additionally, survival analysis was restricted to the population of patients diagnosed in 2010 through 2012 due to the unavailability of synchronous BM data prior to this timeframe.

Clinical and demographic characteristics are presented for the four categories of patients produced by a cross of bevacizumab treatment and SBM status. However, regression models utilized two populations of patients: one population *with* brain metastases, and another representing the *overall* population of NSCLC patients. Patients are therefore described as SBM + or SBM-, indicating presence or absence of SBM, and BEV + or BEV-, indicating presence of a record of bevacizumab treatment versus the absence thereof.

### Treatment patterns

Differences in the use of bevacizumab were investigated across the NSCLC and the NSCLC with SBM populations using descriptive statistics and logistic regression. Descriptive statistics are presented for clinical and demographic characteristics across each of the four populations of patients produced by a cross of SBM and BEV statuses. Univariable logistic regression predicting the use of bevacizumab on the basis of clinical and demographic characteristics is presented to further describe treatment patterns.

Candidate predictors for a multivariable model of bevacizumab prescription included clinical and demographic characteristics, along with prescriptions of other medications that had been used in at least 11 SBM patients who were treated with bevacizumab. Predictors for a multivariable model were selected using the least angle shrinkage and selection operator (LASSO) as implemented by Friedman *et al*. in their software package *glmnet*^18^. The effect of regularization across 81 lambda values from ten thousand to one ten-thousandth were examined using 200-fold validation, and predictors minimizing mean square error across these parameters were selected for inclusion.

### Treatment efficacy

Treatment efficacy in the population of patients diagnosed with brain metastases was evaluated using survival analysis predicting mortality with respect to bevacizumab treatment and patient characteristics. Because several patient characteristics affect the decision to use bevacizumab, propensity score matching was used to account for potential confounding due to treatment decisions. Patients were matched on the basis of propensity to receive bevacizumab treatment using a caliper of 0.1 applied by a greedy nearest-neighbors matching algorithm and a 1:1 ratio of control to treated subjects without replacement. The success of propensity score matching was evaluated by fitting a multivariable logistic regression model to each of the matched and unmatched populations, then visually comparing the distributions of scores and standardized mean differences (Supplementary Figures 2, 3, and 4).

For both the propensity matched and unmatched samples, univariable Cox proportional hazards modeling was used to estimate the survival benefit of bevacizumab use with respect to candidate predictors. Weighted estimation of average hazard ratios was performed where non-proportional survival distributions were found across predictors using methods reported and implemented by Dunkler *et al*. (2018)^19^.

A multivariable Cox proportional hazards model predicting survival was constructed using the same parameters and criteria as those of logistic regression, performed using the *coxnet* implementation provided by Simon *et al*. (2011)^20^. Univariable and adjusted Kaplan-Meier estimates of survival distributions were also included, where adjustment accounted for covariates presented as part of tables in results. The conditional method to balance a population across a set of covariates, described by Therneau *et al*. (2015) and implemented by Kassambra and Kosinski (2018), was used to visualize adjusted curves^21,22^. Additionally, restricted mean survival times (RMST) are presented to explicate survival among each of the two cohorts with or without brain metastases. As a last measure to mitigate potential bias, sensitivity analysis was performed to determine whether observed effects differed when considering the initial event to be BEV prescription rather than primary cancer diagnosis.

### Rigor and reproducibility

All code written to perform analysis is available online as a series of scripts, along with a Makefile to make the entirety of analysis explicit and enable others to replicate this work using data available from SEER-Medicare (Ascha 2019)^23^.

## Results

### Clinical and demographic characteristics

Eighty-one SBM + patients had a record of bevacizumab treatment, while 4,343 patients with SBM did not have such a record. Among those cases without SBM, claims indicating bevacizumab administration were found for 666, and no such evidence was found for 35,197 patients (Table 1). The majority of cases were of adenocarcinoma histology among patients treated with bevacizumab, at 75.3% and 70.7% for SBM + /BEV + and SBM-/BEV + patients, respectively, but only 50.5% and 38.2% of SBM + /BEV- and SBM-/BEV- patients had adenocarcinoma histology. Except for the SBM + /BEV + group, which was comprised of 43.2% males, each of the four populations had an approximately even distribution of males versus females. A greater proportion of SBM + /BEV- patients were of nonwhite race (24.4%) than SBM + /BEV + patients (18.5%), and the age of patients across all four categories was approximately 70 years.

**Table 1:** NSCLC patient characteristics by SBM and bevacizumab treatment status, SEER-Medicare data 2010–2012. An asterisk (*) indicates censoring where fewer than 11 observations were available.

Variable of interest	Bevacizumab		No Bevacizumab	
	SBM	No SBM	SBM	No SBM
N	81	666	4,343	35,196
Age (mean (sd))	69.4 (3.5)	71.1 (4.3)	71.5 (4.4)	72.3 (4.5)
Nonwhite (%)	15 (18.5)	153 (23.0)	1,060 (24.4)	7,755 (22.0)
**Histology**				
Adenocarcinoma	61 (75.3)	471 (71.4)	2,195 (51.1)	13,462 (38.6)
Squamous Cell Carcinoma	*	30 (4.5)	600 (14.0)	10,845 (31.1)
Other	*	159 (24.1)	1,498 (34.9)	10,550 (30.3)
Male (%)	35 (43.2)	336 (50.5)	2,293 (52.8)	18,773 (53.3)
**Stage (%)**				
Stage IA	0 (0.0)	25 (3.8)	0 (0.0)	5,423 (15.4)
Stage IB	0 (0.0)	35 (5.3)	0 (0.0)	4,392 (12.5)
Stage IIA	0 (0.0)	*	0 (0.0)	444 (1.3)
Stage IIB	0 (0.0)	20 (3.0)	0 (0.0)	1,567 (4.5)
Stage IIIA	0 (0.0)	51 (7.7)	0 (0.0)	3,740 (10.6)
Stage IIIB	0 (0.0)	122 (18.5)	0 (0.0)	5,800 (16.5)
Stage IV	81 (100.0)	391 (59.2)	4,343 (100.0)	12,759 (36.3)
Stage Occult	0 (0.0)	*	0 (0.0)	449 (1.3)
Stage Unknown	0 (0.0)	*	0 (0.0)	622 (1.8)
**Bone Metastases (%)**				
None	56 (69.1)	*	2,882 (66.4)	29,917 (85.0)
Unknown	0 (0.0)	*	129 (3.0)	163 (0.5)
Yes	25 (30.9)	198 (29.7)	1,332 (30.7)	5,116 (14.5)
**Liver Metastases (%)**				
None	*	*	3,082 (71.0)	30,352 (86.2)
Unknown	*	*	184 (4.2)	466 (1.3)
Yes	22 (27.2)	152 (22.8)	1,077 (24.8)	4,378 (12.4)
Cranial SRS (%)	70 (86.4)	529 (79.4)	2,631 (60.6)	20,465 (58.1)
Any Radiotherapy (%)	81 (100.0)	610 (91.6)	2,927 (67.4)	22,537 (64.0)
Carboplatin (%)	65 (80.2)	566 (85.0)	860 (19.8)	7459 (21.2)
Cisplatin (%)	*	90 (13.5)	106 (2.4)	1783 (5.1)
Dexamethasone (%)	69 (85.2)	581 (87.2)	1229 (28.3)	10908 (31.0)
Paclitaxel (%)	39 (48.1)	338 (50.8)	421 (9.7)	4682 (13.3)
Pemetrexed (%)	51 (63.0)	501 (75.2)	517 (11.9)	3152 (9.0)

### Treatment Patterns

More than 90% of BEV + cases were found to have some record of radiotherapy treatment, whereas the proportion of BEV- patients undergoing radiotherapy was lower, at about 65%. Pemetrexed treatment was consistently associated with increased odds of bevacizumab treatment; for each of the SBM + and overall NSCLC patient populations, patients receiving pemetrexed had 6.06 (95% CI: 3.45–10.85, p < 0.001) and 8.93 (95% CI: 7.3–11.0, p < 0.001) times the odds of bevacizumab prescription (Supplementary Table 1, Supplementary Table 2).

A great majority of BEV + patients were treated with dexamethasone, ranging from 87.2% to 28.3% in the SBM-/BEV + and SBM-/BEV- populations. Dexamethasone treatment was associated with greater odds of BEV treatment in the SBM + population (OR: 2.9, 95% CI: 1.4–6.4, p = 0.005) and overall patient population (OR: 2.0, 95% CI: 1.5–2.6, p < 0.001), after adjusting for clinical and demographic characteristics.

In both the SBM population and the overall population, treatment with paclitaxel was associated with bevacizumab prescription: adjusting for a variety of clinical and demographic characteristics, SBM + patients treated with paclitaxel had 4.5 times the odds of bevacizumab treatment than their counterparts who were not (95% CI: 2.7–7.6, p < 0.001), and the overall NSCLC population had a corresponding odds ratio of 2.5 (95% CI: 2.1–3.0, p < 0.001).

### Survival analysis

**Figure 1:**
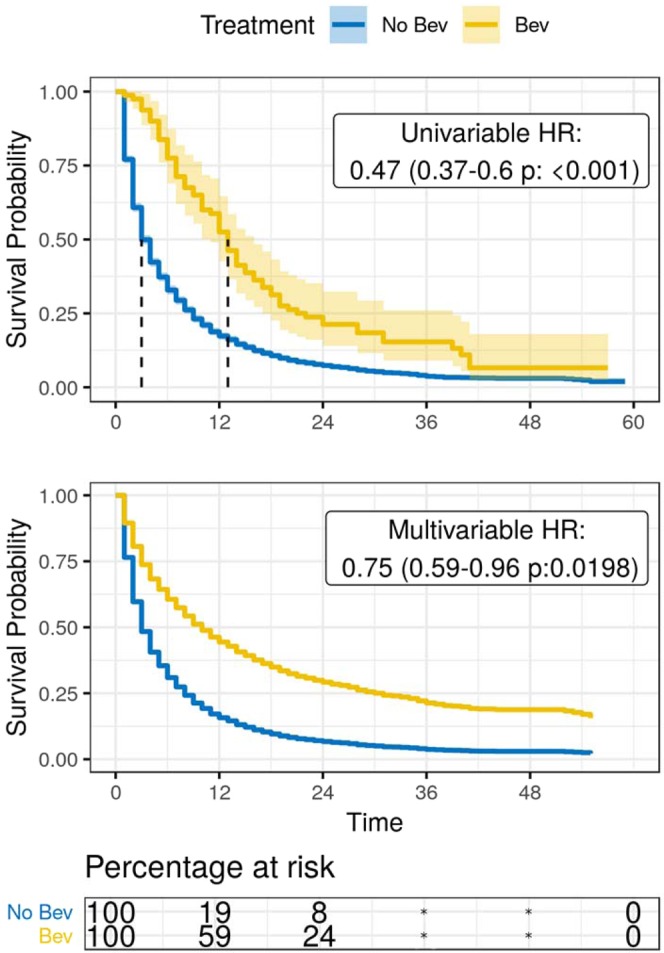
Estimated survival among the NSCLC synchronous brain metastasis population, SEER-Medicare dataset 2010–2012; For NSCLC patients diagnosed with brain metastases, the top panel in this figure shows a Kaplan-Meier estimate of survival for each group of patients treated with and without bevacizumab, and the middle panel shows a conditional survival curve in which each observation is weighted with respect to odds of bevacizumab treatment. At the bottom, a table describes the Kaplan-Meier estimated proportion of patients at risk is provided.

**Figure 2:**
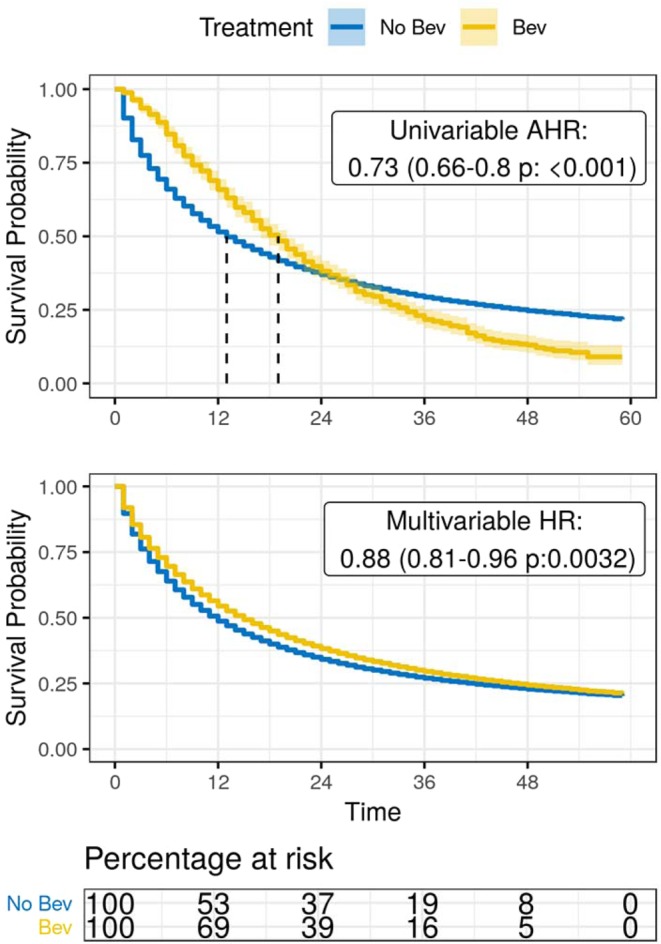
Estimated survival among NSCLC patients, SEER-Medicare dataset 2010–2012; For all NSCLC patients, the top panel in this figure shows a Kaplan-Meier estimate of survival for each group of patients treated with and without bevacizumab, and the middle panel shows a conditional survival curve in which each observation is weighted with respect to odds of bevacizumab treatment. At the bottom, a table of the Kaplan-Meier estimated proportion of patients at risk is provided. Due to non-proportional hazards, weighted estimation of hazard ratios was performed to yield an average hazard ratio of mortality associated with the use of bevacizumab.

Adjusting for clinical and demographic characteristics, bevacizumab treatment was associated with improved survival in the SBM + (HR: 0.75, 95% CI: 0.59–0.96, p: 0.020) and overall population (HR: 0.88, 95% CI: 0.81–0.96, p = 0.003) (Figs. 1, 2); this effect persisted after propensity score matching in both the SBM + (HR: 0.66, 95% CI: 0.47–0.91, p: 0.012) and overall population (HR: 0.71, 95% CI: 0.63–0.80, p < 0.001), and persisted when considering bevacizumab administration as the starting event rather than primary cancer diagnosis (Supplementary Table 3, Table 2). Notably, the distributions of survival times across treatment and control groups in the overall population were non-proportional (p < 0.001), and thus weighted estimation of an average hazard ratio associated with bevacizumab use (AHR: 0.73, 95% CI: 0.66–0.80, p < 0.001) (Supplementary Table 4) is reported. Non-proportionality was further addressed by estimating restricted mean survival time. Restricting to 24 months, which is approximately the time at which surviving proportions intersect (BEV- surviving proportion: 0.37, BEV + surviving proportion: 0.38), this approach confirms the effect observed in weighted estimates: BEV- patients had RMST of 13.8 (95% CI: 13.6, 13.9) whereas corresponding BEV + patients in the overall population showed a RMST of 16.1 (95% CI: 16.0, 17.1), for a RMST ratio of 0.83(95% CI: 0.81, 0.86). Among data elements selected for regression analysis, none had more than 5% of values missing (Supplementary Table 5).

**Table 2:** Hazard ratios of mortality associated with the use of bevacizumab for NSCLC patients overall and those with synchronous brain metastases. Hazard ratios are presented for matched and unmatched populations, as well as for univariable and multivariable models. An asterisk (‘*’) indicates the use of weighted estimation of average hazard ratios, which places greater weight on events occurring sooner after the index date, rather than Cox proportional hazards modeling due to non-proportional hazards. In the ‘Start’ column, ‘Dx’ indicates that the duration under consideration started with primary cancer diagnosis and ‘Rx’ indicates bevacizumab administration date marked the start of survival.

Population	Start	Match	Univariable	Multivariable
NSCLC, Overall	Dx	Unmatched	0.73 (0.66–0.80 p: < 0.001)*	0.88 (0.81–0.96 p:0.0032)
	Dx	Matched	0.71 (0.63–0.80 p: < 0.001)	
	Rx	Unmatched	0.82 (0.75–0.89, p < 0.001)	0.71 (0.65–0.77, p < 0.001)
NSCLC,Synchronous Brain Metastases	Dx	Unmatched	0.47 (0.37–0.60 p: < 0.001)	0.75 (0.59–0.96 p:0.0198)
	Dx	Matched	0.66 (0.47–0.91 p:0.0115)	
	Rx	Unmatched	0.39 (0.31–0.50, p < 0.001)	0.62 (0.48–0.79, p < 0.001)

## Discussion

Bevacizumab was first approved by the FDA for use in 2004, and its targeted mechanism of action was efficacious enough to merit development of biosimilars as soon as 2017^24,25^. Initially approved for metastatic colorectal cancer, non-small cell lung cancer and glioblastoma were added to its indications in 2006 and 2009, respectively^26,27^. Intense research efforts have demonstrated that bevacizumab benefits patients affected by a variety of primary cancers, though its cost poses a barrier to many patients who might otherwise benefit from its use^28^. A precise understanding of its effects and those patients who stand to benefit the most, therefore, is distinctly important to informing treatment decisions. Real-World Data such as Medicare claims may describe the benefit of bevacizumab to NSCLC patients, but significant steps (such as Phase III trials) are necessary to produce regulatory-grade evidence.

Bevacizumab blocks the activity of VEGF-A upregulation in response to e.g. hypoxia-inducible factor 1, thereby directly counteracting the inappropriate angiogenesis that arises from oncometabolic stress^29^. This mechanism has led to its classification as a *cytostatic* agent rather than a *cytotoxic* agent; consequently, it has been suggested that survival endpoints address bevacizumab efficacy more appropriately than objective endpoints that are defined by measurements such as tumor size or spread^30^. Furthermore, the extent of tumor angiogenesis has strong prognostic implications in itself, so the potential benefit of bevacizumab may depend on the extent of disease with respect to angiogenic signalling^31^. Such a hypothesis would be supported by our observations of decreased hazard of mortality among SBM + patients treated with bevacizumab compared to the overall population, and could meaningfully contribute to treatment decisions.

Studies to evaluate the efficacy of bevacizumab most often come in the form of randomized controlled trials and retrospective reviews, but the rise of real-world evidence has recently opened the door to such research using population-level data. Li *et al*. (2019) report on a cohort of 22,258 patients with NSCLC from the US Flatiron Electronic Health Record database, of whom 961 met inclusion criteria^32^. Their examination of the overall population of NSCLC patients treated with bevacizumab revealed no significant differences in overall survival with respect to treatment type, similar to our finding of modest survival benefit resulting from the use of bevacizumab in the Medicare population of NSCLC patients. However, we found that after adjusting for clinical and demographic characteristics among NSCLC patients with synchronous brain metastases, there is a significant survival benefit of bevacizumab such that SBM + /BEV + patients had 0.75 times the hazard of mortality as compared to their SBM + /BEV- counterparts at any given time following primary cancer diagnosis (95% CI: 0.59–0.96, p = 0.020). However, these findings require further investigation in other analyses like Phase III randomized-controlled trials with similar efficacy in BM patient populations.

Concerns regarding a potential association with risk of intracranial hemorrhage have discouraged the use of bevacizumab^33^. Yet, there have been studies of patients with primary brain tumors reporting clinical benefit and no significant increase in intracranial hemorrhage^34^, and a meta-analysis of eight studies covering 8,713 patients demonstrated no increase in the risk of intracranial hemorrhage in patients with brain metastases^35^.

Potential benefits to cranial pressure and hemorrhage, however, must be carefully weighed against the beneficial intracranial effects of anti-VEGF treatments. VEGF-A is a ‘vascular permeability factor’, and bevacizumab reduces vasogenic edema by decreasing blood-brain barrier permeability. Similarly, dexamethasone is used to suppress vascular response to VEGF while also reducing VEGF production^36^. In our population, dexamethasone was used at some point for a majority of SEER-Medicare patients diagnosed with synchronous BM, however, like in previous work, its use was not associated with differences in survival^37^.

In addition to potentially minimizing cerebral edema, bevacizumab has demonstrated significant systemic effects in patients with advanced stage NSCLC. Bernardo *et al*. 2002 reported that patients whose brain metastases respond to this combination therapy also had response in extracerebral sites^38–40^. At the same time, large molecules such as bevacizumab may not cross the blood-brain barrier, so its delivery to brain parenchyma and chemotherapeutic effects on BM may not fully explain the increased survival benefit to patients with BM^41^.

While it reflects a great number of patients, the present observational study is limited with respect to population selection and detail available in these data. The SEER-Medicare linkage allows researchers to examine high-fidelity cancer registry data as it relates to Medicare claims, however, this necessitates a restriction to the senior and elderly population. Though studies using SEER-Medicare are highly generalizable to an older population, conclusions do not reflect the US population at large. Furthermore, these data represent treatment patterns in the US, though there is significant variation in practice patterns across countries. In these claims data, the record of a test that may guide treatment decisions would be present, but the results of such a test would not be available; for example, in NSCLC, where EGFR tests are regularly performed in order to inform decisions to use EGFR-targeted drugs.

Despite these limitations, the availability of population-level cancer and mortality data from a well-established source is unique to the SEER-Medicare dataset, in contrast to other real-world evidence that relies solely on claims to identify death. The present work examines a high volume of data representing more than 40,000 NSCLC patients. This great number of cases was also not subject to usual constraints of high-dimensional data, due to its focus on one medication, with appropriate adjustment for potential confounders.

## Conclusions

In our present study, bevacizumab has demonstrated modest survival benefit among the overall elderly population of NSCLC patients, and is associated with even greater such benefit when used for patients with synchronous BM. Understanding the potential outcomes and usage trends of bevacizumab in large datasets like SEER-Medicare both informs and reflects clinical practice, particularly with respect to the survival benefit of bevacizumab for patients diagnosed with synchronous BM. The potential efficacy of bevacizumab in this setting and our results need further examination and confirmation through phase III randomized controlled trials.

### Ethical approval

This study was approved as exempt of review by the University Hospitals Cleveland Medical Center Institutional Review Board under study number “EM-17–05.””, reviewed and approved by the SEER-Medicare committee as sharing no identifying information and preserving both privacy and confidentiality, and performed and reported in accordance with STROBE guidelines.

## Supplementary information

Supplementary Information
