# Supplementary material for: Bevacizumab for the treatment of non-small cell lung cancer patients with synchronous brain metastases
Source: Sci Rep. 2019 Nov 28;9:17792. doi: 10.1038/s41598-019-54513-3 (PMC6882803; doi:10.1038/s41598-019-54513-3)
Supplement: Supplementary file 1 — Supplementary Information [file 41598_2019_54513_MOESM1_ESM.pdf]

Bevacizumab for the treatment of non-small cell lung cancer patients with synchronous brain metastases

Mustafa S Ascha[1]*, Jacqueline Fang Wang[2]*, Priya Kumthekar, MD[3], Andrew E Sloan, MD[4], Carol Kruchko, BA[5], Jill S Barnholtz-Sloan, PhD[2,6]

1. Center for Clinical Investigation, Department of Population and Quantitative Health Sciences, Case Western Reserve University School of Medicine, Cleveland, Ohio
2. Case Western Reserve University School of Medicine, Cleveland, Ohio
3. Northwestern University Feinberg School of Medicine, Department of Neurology, Evanston, Illinois.
4. Department of Neurosurgery, University Hospitals Cleveland Medical Center, Seidman Cancer Center, and the Case Comprehensive Cancer Center, Cleveland, Ohio
5. Central Brain Tumor Registry of the United States, Hinsdale, Illinois
6. Department of Population and Quantitative Health Sciences, Case Western Reserve University School of Medicine, Cleveland, Ohio

*Joint first authorship

Abstract: 200 words

Manuscript: 3189 words

Running head: Bevacizumab for non-small cell lung cancer brain metastases

Keywords: brain metastases; non-small cell lung cancer; bevacizumab; SEER-Medicare

Corresponding author:
Jill Barnholtz-Sloan, PhD
Sally S. Morley Designated Professor in Brain Tumor Research
Case Comprehensive Cancer Center, Institute for Computational Biology
Case Western Reserve University School of Medicine
2-526 Wolstein Research Bldg, 2103 Cornell Rd
Cleveland, Ohio 44106-7295
216-368-1506

jsb42@case.edu

Supplementary Table 1: Odds of bevacizumab prescription among NSCLC synchronous brain metastases patients diagnosed in the years 2010 through 2012; Reference levels include 65 to 70 years of age, non-adenocarcinoma histology, no medication administered, female sex, and no surgical management. For categories with more than two possible values, asterisks are used to denote reference levels.

	Univariable	Multivariable
Age: 65 to 70	*	*
Age: 70 to 74	0.66 (0.39-1.08, p: 0.1107)	0.63 (0.36-1.05, p: 0.0860)
Age: 75+	0.25 (0.09-0.53, p: 0.0012)	0.31 (0.12-0.70, p: 0.0076)
Cisplatin	5.17 (2.35-10.13, p < 0.001)	1.46 (0.63-3.03, p: 0.3417)
Dexamethasone	13.74 (7.69-26.80, p < 0.001)	2.92 (1.42-6.36, p: 0.0048)
Adenocarcinoma	2.79 (1.70-4.76, p: 0.0001)	1.63 (0.94-2.90, p: 0.0869)
Male	0.66 (0.42-1.03, p: 0.0712)	0.74 (0.46-1.20, p: 0.2235)
Paclitaxel	7.92 (4.99-12.49, p < 0.001)	4.47 (2.65-7.56, p < 0.001)
Pemetrexed	13.93 (8.73-22.71, p < 0.001)	6.06 (3.45-10.85, p < 0.001)

Supplementary Table 2: Odds of bevacizumab prescription among all NSCLC patients diagnosed in the years 2010 through 2012. Reference levels include 65 to 70 years of age, stage I disease, non-adenocarcinoma histology, no medication administered, white race, female sex, and no surgical management. For categories with more than two possible values, asterisks are used to denote reference levels.

	Univariable	Multivariable
Age: 65 to 70	*	*
Age: 70 to 74	0.72 (0.61-0.85, p < 0.001)	0.77 (0.64-0.92, p: 0.0038)
Age: 75+	0.51 (0.42-0.62, p < 0.001)	0.76 (0.62-0.94, p: 0.0107)
Stage I	*	*
Stage II	1.95 (1.19-3.10, p: 0.0059)	0.81 (0.49-1.32, p: 0.4093)
Stage III	2.98 (2.23-4.03, p < 0.001)	0.91 (0.65-1.28, p: 0.5745)
Stage IV	4.61 (3.55-6.10, p < 0.001)	1.49 (1.09-2.08, p: 0.0151)
Stage Occult	2.55 (1.06-5.24, p: 0.0200)	1.49 (0.58-3.29, p: 0.3646)
Stage Unknown	1.16 (0.35-2.84, p: 0.7695)	0.54 (0.16-1.38, p: 0.2473)
Adenocarcinoma	3.74 (3.19-4.40, p < 0.001)	1.68 (1.41-2.01, p < 0.001)
Carboplatin	20.27 (16.66-24.89, p < 0.001)	2.56 (1.96-3.37, p < 0.001)
Dexamethasone	15.03 (12.18-18.75, p < 0.001)	2.00 (1.53-2.64, p < 0.001)
Paclitaxel	6.81 (5.88-7.89, p < 0.001)	2.47 (2.07-2.95, p < 0.001)
Pemetrexed	28.01 (23.72-33.21, p < 0.001)	8.93 (7.27-11.00, p < 0.001)
Non-White	1.01 (0.84-1.19, p: 0.9523)	1.19 (0.98-1.43, p: 0.0769)
Male	0.86 (0.74-1.00, p: 0.0445)	0.88 (0.75-1.03, p: 0.1078)
Surgery	0.46 (0.37-0.56, p < 0.001)	0.94 (0.72-1.21, p: 0.6347)

	Univariable	Multivariable
Bevacizumab	0.47 (0.37-0.60, p < 0.001)	0.75 (0.59-0.96, p:0.0198)
Age: 65 to 70	*	*
Age: 70 to 74	1.10 (1.02-1.19, p: 0.0096)	1.14 (1.06-1.23, p < 0.001)
Age: 75+	1.31 (1.20-1.43, p < 0.001)	1.26 (1.16-1.37, p < 0.001)
Metropolitan	0.93 (0.85-1.02, p: 0.1120)	0.89 (0.81-0.97, p: 0.0092)
Non-White	0.88 (0.82-0.95, p: 0.0016)	0.82 (0.76-0.89, p < 0.001)
Adenocarcinoma	0.74 (0.70-0.79, p < 0.001)	0.80 (0.74-0.85, p < 0.001)
Male	1.14 (1.06-1.21, p < 0.001)	1.13 (1.06-1.20, p < 0.001)
Carboplatin	0.57 (0.53-0.61, p < 0.001)	0.77 (0.67-0.88, p < 0.001)
Cisplatin	0.57 (0.47-0.69, p < 0.001)	0.67 (0.55-0.82, p < 0.001)
Dexamethasone	0.63 (0.59-0.68, p < 0.001)	0.96 (0.87-1.06, p: 0.4586)
Paclitaxel	0.62 (0.56-0.69, p < 0.001)	0.78 (0.68-0.89, p < 0.001)
Pemetrexed	0.50 (0.46-0.55, p < 0.001)	0.69 (0.61-0.78, p < 0.001)

Supplementary Table 3: Hazard ratios of mortality among the NSCLC synchronous brain metastases patients, SEER-Medicare dataset 2010-2012. When considering only patients diagnosed with brain metastases during staging workup for primary cancer, the above shows ratios for hazard of mortality. After adjusting for clinical and demographic characteristics, patients with brain metastases who were treated with bevacizumab were found to have 0.75 times the hazard of mortality at any given time, compared to the corresponding untreated population. For categories with more than two possible values, asterisks are used to denote reference levels.

Supplementary Table 4: Hazard ratios of mortality in the overall NSCLC population, SEER-Medicare dataset 2010-2012. Reference levels include 65 to 70 years of age, stage I disease, non-adenocarcinoma histology, no medication administered, white race, female sex, non-metropolitan diagnosing or treating facility, and no surgical management.

	Univariable	Multivariable
Bevacizumab	0.73 (0.66-0.80, p < 0.001)	0.88 (0.81-0.96, p:0.0032)
Age: 65 to 70	*	*
Age: 70 to 74	1.10 (1.07-1.13, p < 0.001)	1.12 (1.08-1.15, p < 0.001)
Age: 75+	1.21 (1.18-1.25, p < 0.001)	1.25 (1.22-1.29, p < 0.001)
Stage I	*	*
Stage II	1.84 (1.72-1.96, p < 0.001)	2.00 (1.87-2.13, p < 0.001)
Stage III	3.10 (2.98-3.22, p < 0.001)	3.48 (3.34-3.63, p < 0.001)
Stage IV	5.96 (5.74-6.19, p < 0.001)	6.88 (6.63-7.15, p < 0.001)
Stage Occult	2.97 (2.64-3.35, p < 0.001)	3.07 (2.72-3.46, p < 0.001)
Stage Unknown	3.75 (3.39-4.14, p < 0.001)	3.96 (3.58-4.37, p < 0.001)
Metropolitan	0.86 (0.84-0.89, p < 0.001)	0.86 (0.83-0.89, p < 0.001)
Non-White	1.04 (1.01-1.07, p: 0.0194)	0.93 (0.90-0.96, p < 0.001)
Adenocarcinoma	0.96 (0.94-0.99, p: 0.0017)	0.88 (0.86-0.90, p < 0.001)
Male	1.28 (1.25-1.31, p < 0.001)	1.25 (1.22-1.28, p < 0.001)
Carboplatin	1.08 (1.05-1.11, p < 0.001)	0.89 (0.85-0.94, p < 0.001)
Cisplatin	0.74 (0.70-0.79, p < 0.001)	0.72 (0.68-0.76, p < 0.001)
Dexamethasone	0.95 (0.93-0.97, p < 0.001)	0.93 (0.89-0.96, p < 0.001)
Paclitaxel	1.05 (1.02-1.09, p: 0.0024)	0.93 (0.88-0.97, p:0.0014)
Pemetrexed	1.00 (0.97-1.04, p: 0.8774)	0.81 (0.77-0.85, p < 0.001)

Supplementary Table 5: Proportions of values missing and imputed

Data element	Proportion missing (%)
Age	0%
Bone Metastases	4.73%
Liver Metastases	4.84%
Brain Metastases	5.00%
Derived AJCC Stage	0.00%
Urban/Rural classification	0.02%
Race	0.00%
Histology	0.00%
Sex	0.00%

Supplementary Figure 1: Population selection diagram

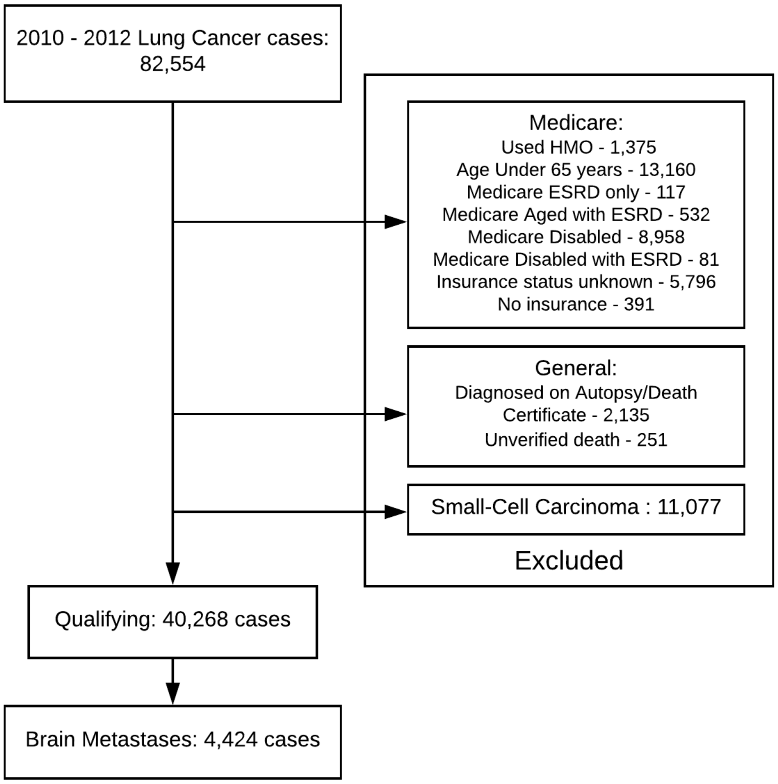

Supplementary Figure 1 describes the population selection procedure required to identify Non-Small Cell Carcinoma (NSCLC) patients with brain metastases (BM). Small-Cell Lung Carcinoma patients were excluded, as were patients who did not meet Medicare criteria and those patients who were diagnosed on autopsy or had unverified death.

Supplementary Figure 2: Propensity score histograms for the SBM population

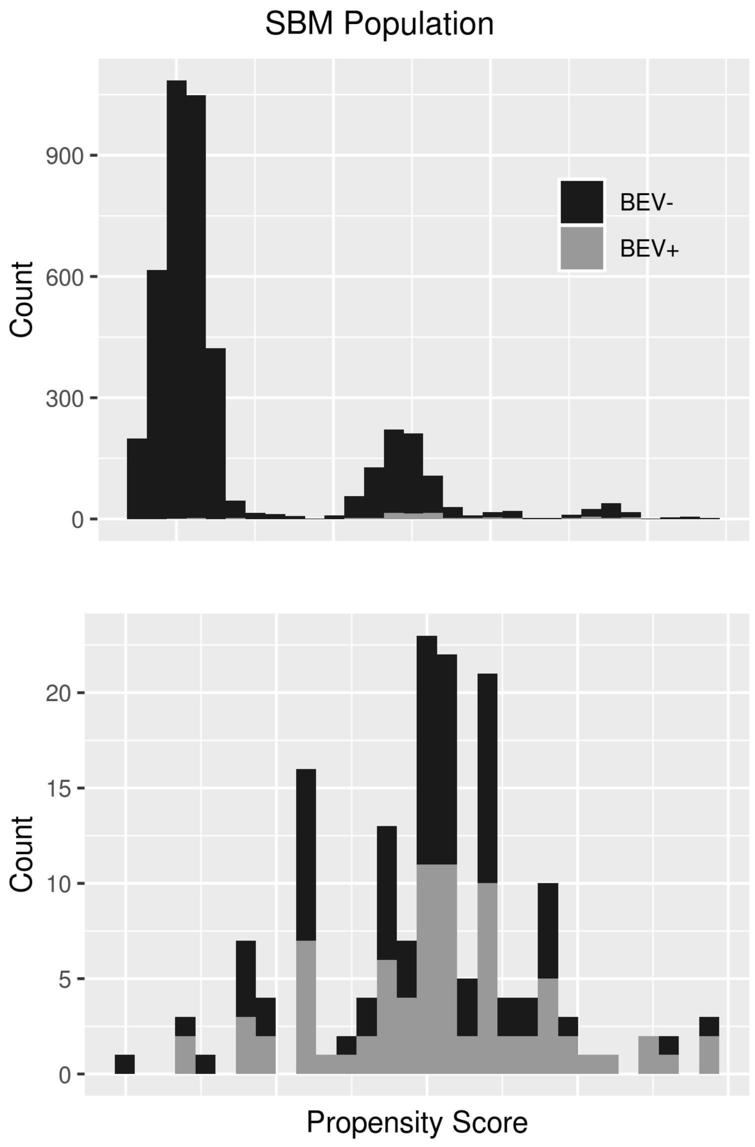

Supplementary Figure 2 shows the distributions of predicted propensity to receive bevacizumab treatment for each of the unmatched (top) and matched (bottom) populations of patients with synchronous brain metastases (SBM).

Supplementary Figure 3: Propensity score histograms for the overall population

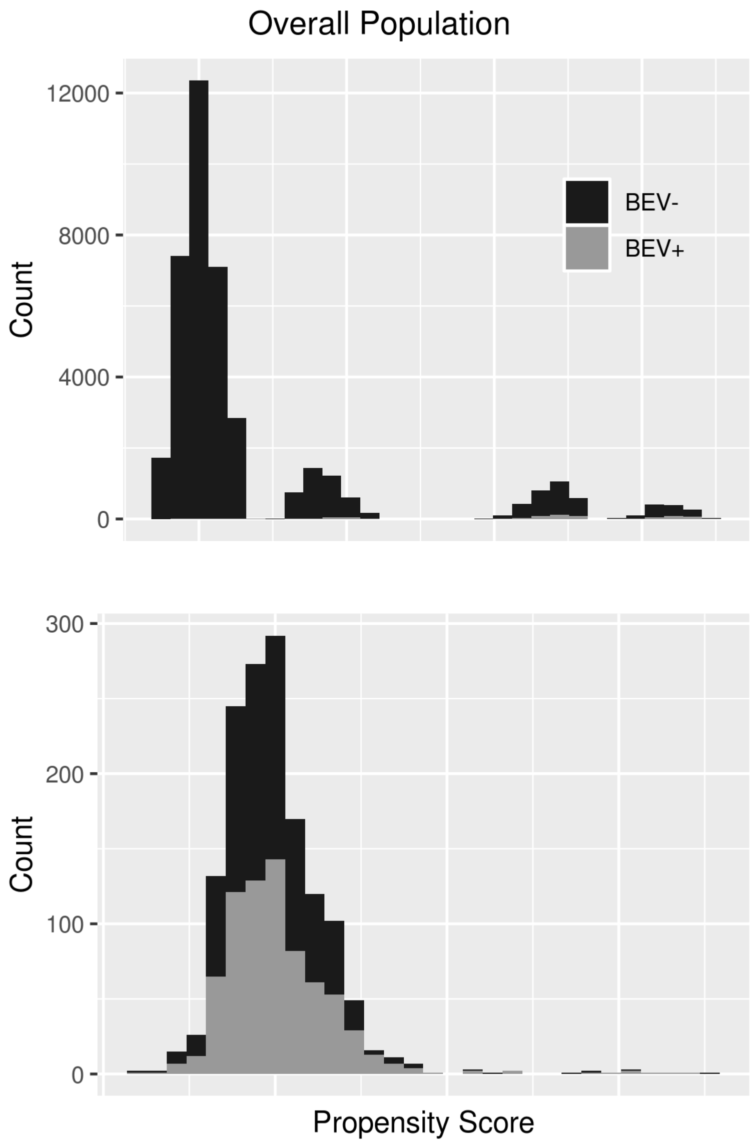

Supplementary Figure 3 shows the distributions of predicted propensity to receive bevacizumab treatment for each of the unmatched (top) and matched (bottom) populations of all patients who met inclusion criteria.

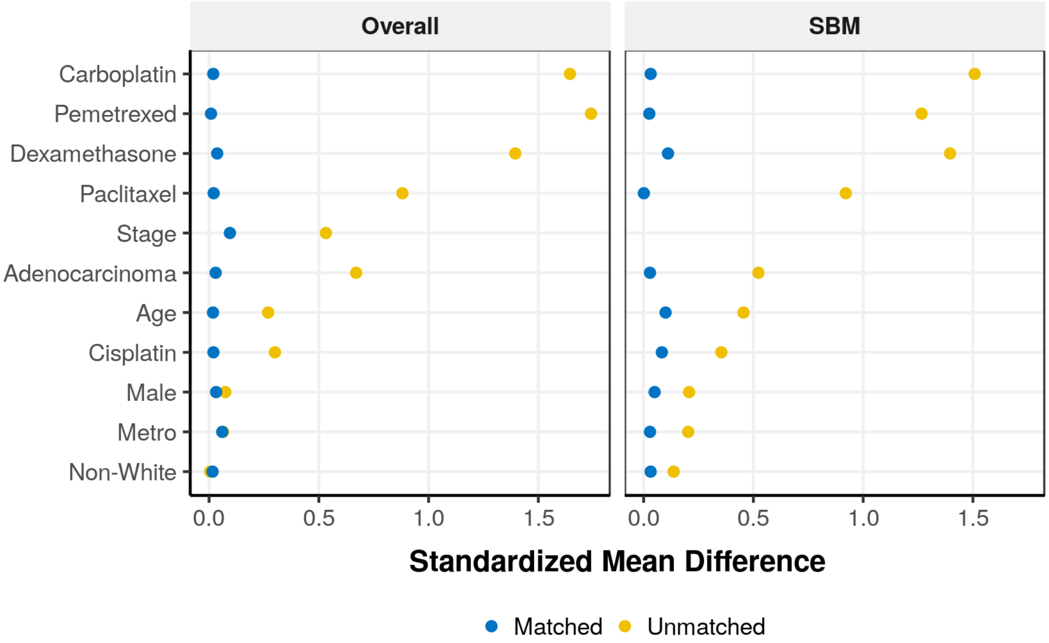

Supplementary Figure 4 depicts the standardized mean differences of potential covariates across matched versus unmatched populations for each of the Synchronous Brain Metastases (SBM) and overall populations. Points shown in blue reflect the matched populations, while those in gold reflect the unmatched populations.
